# Effects of Vitamin K Administration in Correcting Coagulopathy in Patients with Liver Cirrhosis: Retrospective Clinical Study

**DOI:** 10.3390/clinpract15100188

**Published:** 2025-10-18

**Authors:** Magdalena Lixandru, Maniu Ionela, Florin Grosu

**Affiliations:** 1 Faculty of Medicine, “Lucian Blaga” University of Sibiu, 550025 Sibiu, Romania; 2 Sibiu County Emergency Clinical Hospital, 550025 Sibiu, Romania; florin.grosu@ulbsibiu.ro; 3 Valcea County Emergency Hospital, 240595 Râmnicu Vâlcea, Romania; 4 Research Center in Informatics and Information Technology, Mathematics and Informatics Department, Faculty of Sciences, “Lucian Blaga” University of Sibiu, 550025 Sibiu, Romania; 5 Pediatric Clinical Research Department, Pediatric Clinical Hospital Sibiu, 550166 Sibiu, Romania

**Keywords:** liver cirrhosis, vitamin K, coagulopathy, INR, prothrombin time

## Abstract

**Background/Objectives:** Coagulopathy is a common complication of liver cirrhosis, partly due to impaired synthesis of vitamin K-dependent coagulation factors. Despite its frequent use, the efficacy of vitamin K in this setting remains uncertain. This study aimed to evaluate the effect of vitamin K administration on coagulation parameters in patients with cirrhosis. **Methods:** We performed a retrospective study of 122 cirrhotic patients hospitalized between 2020 and 2024, who received vitamin K for coagulopathy correction. Coagulation and liver function parameters were monitored over time. **Results:** An early and progressive improvement in INR values was observed, in a subset of patients, following vitamin K administration. INR values across time points were strongly correlated, while only weak associations were observed with bilirubin levels. These findings support a selective therapeutic effect, rather than universal efficacy. **Discussion:** Despite current guidelines discouraging routine vitamin K use in cirrhosis, our findings suggest that selected patients—particularly those with reversible deficiency—may benefit from supplementation. The observed decrease in INR values supports a targeted, context-based approach rather than empirical management. **Conclusions:** However, due to the retrospective design and absence of a control group, the observed improvements cannot be conclusively attributed to vitamin K administration. Vitamin K may improve coagulation in cirrhotic patients with reversible deficiency, but its efficacy is not universal. Its use should be individualized and guided by clinical and biochemical context, as part of a broader treatment strategy.

## 1. Introduction

Liver cirrhosis represents the final stage in the progression of chronic liver diseases, characterized by extensive fibrosis, architectural distortion of the liver, and impaired hepatocellular function. Globally, it remains a major contributor to morbidity and mortality, accounting for over one million deaths annually. The causes of cirrhosis are diverse, including chronic infections with hepatitis B and C viruses, excessive alcohol intake, non-alcoholic steatohepatitis (NASH), and other chronic liver conditions. Despite therapeutic advances, particularly in antiviral treatment, cirrhosis continues to pose significant challenges in clinical management and is often associated with poor outcomes [1,2,3].

Coagulopathy is one of the most clinically significant complications of cirrhosis, reflecting the liver’s impaired synthetic function and resulting in a complex imbalance between pro- and anticoagulant forces. Reduced hepatic production of clotting factors—particularly the vitamin K–dependent ones (factors II, VII, IX, and X)—contributes substantially to this disturbance. Moreover, as demonstrated by thromboelastometry studies, patients with cirrhosis often exhibit abnormal clot formation and regulation of fibrinolysis. While elevated INR is commonly used to assess coagulation status in cirrhotic patients, it does not accurately predict bleeding risk in this population, complicating clinical assessment [4,5]. Although cirrhosis is often associated with bleeding, it also confers a heightened risk of thrombosis, reflecting a complex state of rebalanced hemostasis. This paradox stems in part from reduced hepatic synthesis of both procoagulant and anticoagulant proteins, particularly protein C and protein S, which are essential for regulating thrombin generation. While INR and PT are commonly elevated in cirrhotic patients, these measures fail to accurately predict bleeding risk and should not be interpreted in isolation [4,5,6].

Treatment of coagulopathy in cirrhosis is essential in clinical management. Fresh frozen plasma (FFP) and coagulation factor concentrates are frequently administered to restore hemostatic balance. However, both options present notable limitations: FFP transfusion carries risks of transfusion reactions and pathogen transmission, and both FFP and concentrates require careful dosing due to variable pharmacokinetics in cirrhotic patients and lack of standardized protocols [6,7,8]. In this context, administration of vitamin K has been proposed to improve hepatic production of vitamin K–dependent clotting factors and re-establish hemostatic balance. Vitamin K1 (phytomenadione) is essential for the γ-carboxylation of coagulation factors II, VII, IX, and X, enabling their biological activity. Clinical and experimental evidence indicates that supplementation with vitamin K may enhance carboxylation efficiency and improve coagulation profiles in patients with liver disease [9,10].

However, administering vitamin K to patients with liver cirrhosis raises several clinical challenges. Individual responses are highly variable, particularly in advanced disease, where impaired hepatic metabolism limits the liver’s ability to carboxylate vitamin K–dependent clotting factors even after supplementation. Thus, despite adequate dosing, many patients do not achieve meaningful improvements in coagulation profiles [11,12]. Intravenous vitamin K administration in cirrhotic patients also carries notable risks. Although hypersensitivity and anaphylactoid reactions are rare, documented cases—including severe hypotension and shock—necessitate caution. Moreover, several clinical studies have demonstrated that even correctly dosed vitamin K may not lead to a significant or sustained correction of INR, due to impaired hepatic processing in advanced liver disease. Hence, careful dosing and vigilant monitoring of coagulation parameters and patient safety are essential [2,13,14,15].

The primary objective of this study is to evaluate the effectiveness and safety of intravenous vitamin K in correcting coagulopathy among patients with liver cirrhosis. The study analyzes changes in coagulation parameters—including INR, prothrombin time, and vitamin K–dependent clotting factor levels—measured before, during, and after treatment. Findings from this study may help refine treatment protocols and support evidence-based use of vitamin K in cirrhotic coagulopathy management.

## 2. Materials and Methods

This retrospective observational study was conducted in the Internal Medicine Department of Vâlcea County Emergency Hospital between January 2020 and December 2024. The study included adult patients (aged 18–85) diagnosed with advanced chronic liver disease of any etiology (viral, alcoholic, or NASH), confirmed through clinical examination and paraclinical investigations (laboratory and imaging evaluation). All patients had grade I–III esophageal varices confirmed by endoscopy and received either oral or intravenous vitamin K for the correction of coagulopathy or the prevention of bleeding. Vitamin K was administered daily during hospitalization. The intravenous pharmaceutical form was ampoule (Phytomenadione 10 mg/mL) in 86 patients, while the other 36 patients received oral vitamin K (Konakion 10 mg). The laboratory methods used for the determination of prothrombin time (PT), INR, activated partial thromboplastin time (APTT) and fibrinogen were the coagulometric-optical method. As for bilirubin and its fractions, the method used was spectrophotometry. Inclusion required documented baseline coagulation tests (INR and prothrombin time) and a MELD score of ≥15 at the initiation of vitamin K therapy. Patients were hospitalized in the intensive care or gastroenterology units for complications related to liver cirrhosis, and only those with complete medical records (electronic or archived) and documented upper gastrointestinal bleeding or high bleeding risk were included. During hospitalization, liver function and coagulation parameters were periodically monitored. Only patients with sufficient follow-up data to evaluate treatment response and clinical progression were included in the final analysis.

Exclusion criteria were age under 18 or over 85; acute viral hepatitis or unstable acute liver disease; hepatic or extrahepatic malignancies; known hereditary coagulation disorders (e.g., hemophilia, von Willebrand disease); severe renal impairment (serum creatinine > 3 mg/dL or chronic dialysis); severe allergies or a history of anaphylaxis to vitamin K; advanced cardiovascular disease or recent myocardial infarction; stage IV COPD; recent liver transplantation or active listing for transplant; and non-adherence risks such as active alcohol or substance abuse. Patients with terminal comorbidities likely to interfere with outcome assessment were also excluded.

Continuous variables were reported as median values with interquartile ranges (IQR: (25th and 75th percentiles), and categorical variables as frequencies and percentages. Changes in biological parameters before and after vitamin K administration were analyzed using non-parametric tests for paired data (Friedman or Wilcoxon tests). Correlations between variables were assessed using Spearman’s rank correlation coefficient. A *p*-value < 0.05 was considered statistically significant.

This study was approved by the Scientific Research Ethics Committee of the “Lucian Blaga” University of Sibiu through ethical opinion number 3 of 17 January 2025. At the time of data collection, written informed consent was obtained from all participants, explicitly authorizing the use of their medical information for scientific research purposes.

## 3. Results

This study included 122 patients diagnosed with liver cirrhosis, over the five-year study period (2020–2024), the number of patients included increased progressively, with the highest proportion recorded in 2024 (32.79%). The cohort was predominantly male (61.48%), and residential distribution was evenly distributed between urban and rural settings (50% each), suggesting a similar incidence of cirrhosis across geographic areas (Table 1).

Regarding the distribution of cirrhosis etiologies, alcohol-related cirrhosis was the most common cause overall (47.54%), followed by cryptogenic (21.31%) and viral etiologies (17.21%). MASLD accounted for a smaller proportion (13.93%). The etiologic pattern was consistent between rural and urban populations. According to the Child–Pugh classification, more than half of the patients (51.64%) were in Class A, indicating compensated liver function. This is a relevant consideration when interpreting the response to vitamin K, given that hepatic synthetic capacity strongly influences coagulation factor production (Table 1).

**Figure 1:**
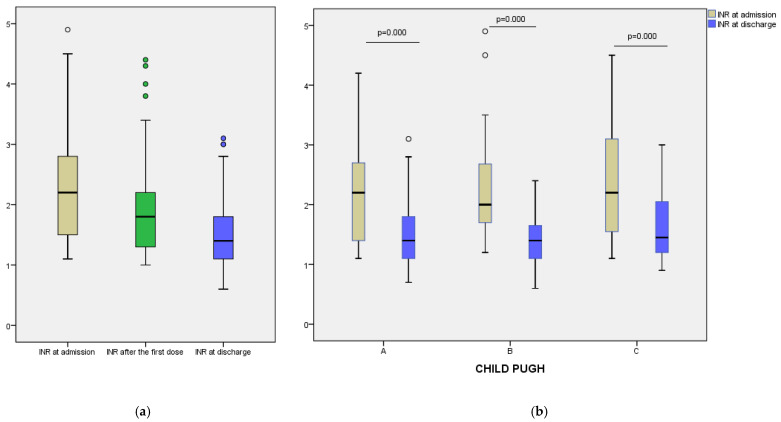
(**a**) INR values at admission, after the first dose of vitamin K and at discharge; (**b**) INR values at admission and at discharge, stratified by Child–Pugh categories.

**Figure 2:**
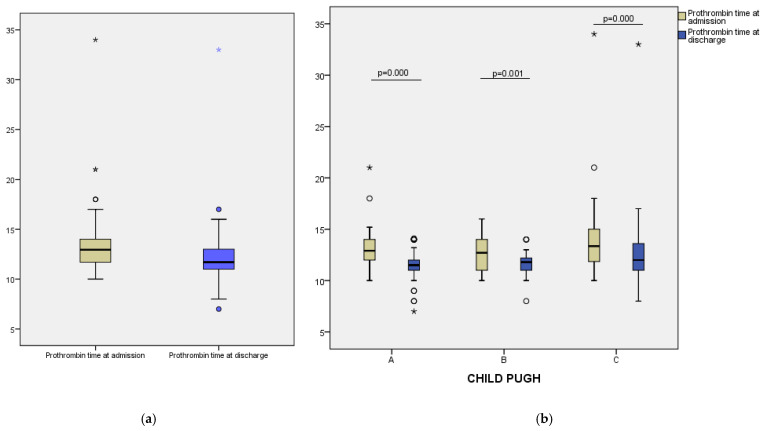
(**a**) Changes in prothrombin time between admission and discharge; (**b**) changes in prothrombin time (outliers values are marked with circle and *) between admission and discharge stratified by Child–Pugh categories.

**Figure 3:**
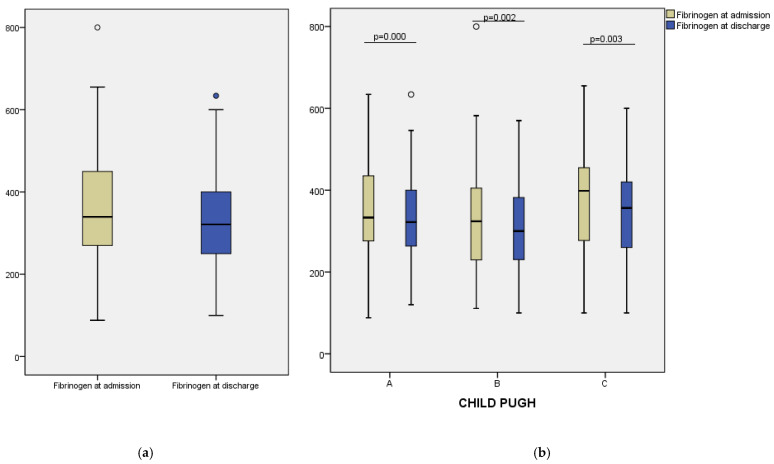
(**a**) Fibrinogen levels at admission and discharge; (**b**) Fibrinogen levels at admission and discharge stratified by Child–Pugh categories.

A progressive reduction in INR values was observed over the course of hospitalization, with a median decrease from 2.20 (IQR: 1.50–2.80) at admission to 1.80 (IQR: 1.30–2.20) after the first dose of vitamin K (24 h), and further to 1.40 (IQR: 1.10–1.80) at discharge (Figure 1a). The Friedman test revealed a statistically significant difference between all three time points (*p* < 0.001), indicating a consistent therapeutic response across the study group. Similarly, prothrombin time decreased from a median of 12.95 (IQR: 11.70–14.00) seconds at admission to 11.70 (IQR:11.00–13.00) seconds at discharge (*p* = 0.000) (Figure 2a). These results suggest that vitamin K administration contributed to improved coagulation status in most patients. Fibrinogen levels showed a modest decline during hospitalization, from a median of 339.50 mg/dL at admission to 321.00 mg/dL at discharge (*p* = 0.000) (Figure 3a). This decrease may reflect reduced inflammatory activity or partial normalization of coagulopathy mechanisms.

To assess differential responses to vitamin K supplementation, we analyzed changes in coagulation parameters across Child–Pugh classes. In Class A patients, median INR decreased from 2.20 at admission to 1.80 after 24 h, and further to 1.40 at discharge. A similar trend was noted in Class B, with INR values declining from 2.00 to 1.80 and reaching 1.40. For Class C, median INR fell from 2.20 to 1.95 at 24 h and to 1.45 at discharge (Figure 1b). Prothrombin time also improved across all groups: from 12.90 to 11.50 s in Class A, from 12.70 to 11.80 s in Class B, and from 13.35 to 12.00 s in Class C (Figure 2b). Median fibrinogen levels showed a modest decrease at discharge in each class—333.00 to 322.00 mg/dL (Class A), 324.00 to 300.00 mg/dL (Class B), and 398.50 to 356.50 mg/dL (Class C) (Figure 3b).

**Figure 4:**
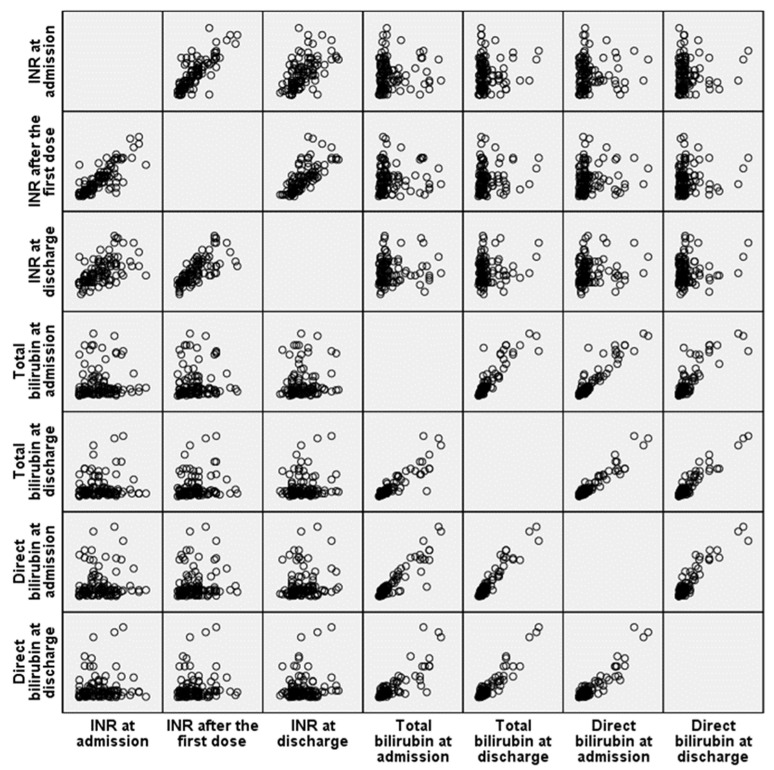
Scatter plot matrix illustrating Spearman’s correlation between INR values (at admission, 24 h, and discharge) and bilirubin levels (total and direct) at admission and discharge.

Spearman’s rank correlation analysis was performed to explore the relationship between INR values and both total and direct bilirubin levels (Figure 4). A strong and statistically significant positive correlation was found between INR values at all three time points (admission vs. 24 h: r = 0.804; 24 h vs. discharge: r = 0.808; *p* < 0.001), indicating a consistent and predictable response to vitamin therapy within individual patients. In contrast, only weak but statistically significant correlations were observed between coagulation and bilirubin levels (INR after 24 h and total bilirubin at admission: r = 0.205; *p* = 0.024; INR at discharge and total bilirubin at admission: r = 0.201; *p* = 0.027; INR after 24 h and direct bilirubin at admission: r = 0.234; *p* = 0.009). No significant correlations were found between INR values and bilirubin levels at discharge, suggesting that the initial cholestatic impact on coagulation may diminish with treatment and clinical improvement.

**Figure 5:**
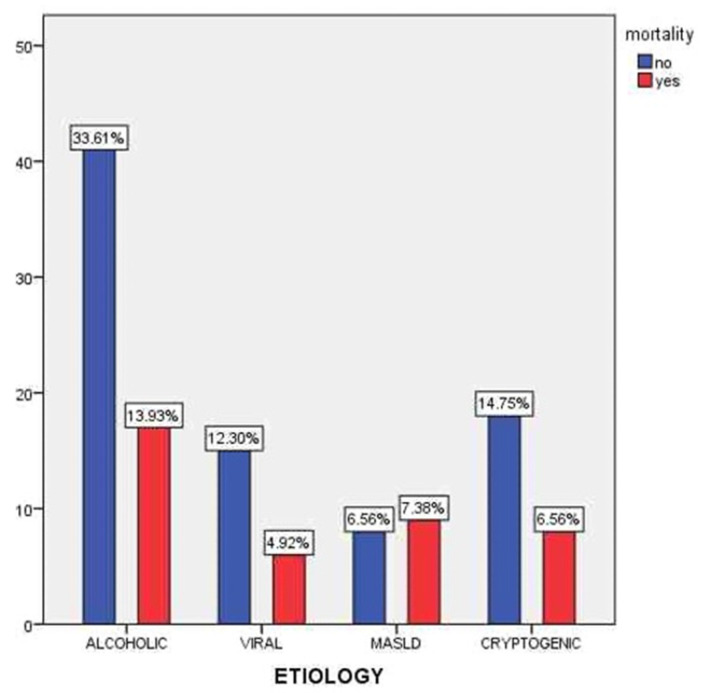
Mortality distribution by cirrhosis etiology.

Figure 5 presents the distribution of mortality across different etiologies of liver cirrhosis. The highest number of deaths was observed among patients with alcohol-related cirrhosis (13.93%), followed by cryptogenic cirrhosis (6.56%) and MASLD (7.38%). Although alcoholic cirrhosis was the most prevalent etiology (47.54% of the cohort), the associated mortality rate was notably elevated, likely due to a more severe clinical course, frequent comorbidities, and poor adherence to medical recommendations. Despite representing a numerically smaller subgroup, patients with MASLD exhibited a relatively high mortality rate, which may reflect the often-silent progression of metabolic liver disease and delayed diagnosis. Conversely, viral cirrhosis showed the lowest mortality (4.92%), potentially due to the beneficial effects of antiviral therapy and better clinical follow-up.

Throughout hospitalization, dynamic changes in liver function markers were observed, suggesting a modest but favorable clinical evolution. The median total bilirubin level decreased from 2.75 mg/dL at admission to 2.10 mg/dL at discharge, while direct bilirubin values declined from 1.40 mg/dL to 1.10 mg/dL, indicating partial improvement in cholestasis and hepatocellular clearance. Serum albumin, a key indicator of hepatic synthetic capacity, rose from a median of 2.80 g/dL to 3.50 g/dL, reflecting improved protein synthesis and nutritional status. Cholinesterase levels increased during hospitalization, from a median value of 3588.50 U/L at admission to 4336.00 U/L at discharge, suggesting a modest trend toward improved hepatic synthetic function. Transaminase activity showed a mild reduction: AST (aspartate aminotransferase) decreased from 47.00 U/L to 41.00 U/L, and ALT (alanine aminotransferase) from 44.00 U/L to 40.00 U/L, suggesting attenuation of hepatocellular injury. Additionally, GGT levels declined from 48.00 U/L to 31.50 U/L, supporting a trend toward reduced cholestatic stress. Although modest, these cumulative changes reflect a general trend toward stabilization or mild recovery of liver function during the observation period.

At 24 h after vitamin K administration, 53 out of 122 patients (43.4%) met the predefined response criterion (as used by Rivosecchi et al. [15]), defined as either a ≥30% reduction in INR compared to baseline or achieving an INR ≤ 1.5. By discharge, the number of responders increased to 91 patients (74.6%), suggesting a delayed yet clinically relevant improvement in coagulation parameters in a larger subset of patients. This progressive increase supports the presence of a partial, time-dependent effect of vitamin K in selected cirrhotic individuals (Table 2).

## 4. Discussion

The consistent decrease in INR values following intravenous vitamin K administration observed in this cohort implies that, despite advanced liver disease, a subset of cirrhotic patients may retain sufficient functional hepatocellular reserve to facilitate partial restoration of vitamin K–dependent coagulation pathways. This finding invites a critical re-examination of current clinical assumptions regarding the irreversibility of coagulopathy in cirrhosis and underscores the need to contextualize such therapeutic responses.

In our cohort, we observed a statistically significant reduction in INR values following vitamin K administration, with a progressive decrease from a median of 2.20 at admission to 1.80 after 24 h, and 1.40 at discharge. This dynamic response indicates that at least a subset of patients with liver cirrhosis may retain sufficient hepatic synthetic function to respond to vitamin K therapy. Our findings contrast with some of the previously published data suggesting that vitamin K has limited efficacy in improving coagulation in cirrhotic patients. By contrast, Saja et al. investigated the effect of a single 10 mg subcutaneous dose of vitamin K1 in a cohort of patients with liver disease, including 24 with cirrhosis, and reported no significant changes in PT, fibrinogen, factor VII, protein C, or protein S after 72 h. Their findings suggest that, in many cases, prolonged PT may not be driven by vitamin K deficiency. The discrepancy between our results and theirs may stem from differences in patient selection, route of administration, disease severity, or baseline hepatic reserve [16].

Our findings, which show a favorable INR response in a subset of patients, which could be correlated with vitamin K administration, should be interpreted in light of current international guidelines and existing literature. The European Association for the Study of the Liver (EASL) explicitly discourages routine administration of vitamin K in patients with cirrhosis, noting that prolonged prothrombin time (PT) in this population primarily reflects impaired hepatic synthesis of both vitamin K–dependent and –independent clotting factors, rather than a reversible vitamin deficiency [17]. This is consistent with other studies indicating that the standard correction of INR with vitamin K is often ineffective in the absence of proven deficiency. Tripodi and Mannucci emphasize that despite elevated INR values, patients with cirrhosis may exist in a rebalanced hemostatic state and are not necessarily prone to bleeding unless additional triggers are present [11]. Similarly, Northup and Caldwell argue that conventional coagulation tests, including INR, are poor predictors of bleeding risk in cirrhosis, and that vitamin K administration should not be reflexively applied based on these values alone [7]. These perspectives support a more individualized, evidence-based approach to the use of vitamin K, as empirical supplementation may not yield clinical benefit in many cirrhotic patients.

In light of our findings, several recent studies reinforce the notion that vitamin K-based interventions may not be uniformly effective in cirrhotic patients. Jin et al. emphasize that routine use of intravenous phytonadione should be avoided unless there is clear evidence of vitamin K deficiency, citing its limited impact on coagulation parameters in advanced liver disease [18]. This aligns with our data, which showed a decrease in INR values in a subset of patients, suggesting that hepatic functional reserve plays an important role in treatment response.

Similarly, Meyer et al. retrospectively analyzed 276 hospitalized cirrhotic patients and found that vitamin K administration did not significantly influence INR changes or reduce bleeding events. Instead, improvements were associated with intensive care support, transfusions, and higher baseline INR values [19]. Complementing these findings, Al Sulaiman et al. also reported only modest INR correction following vitamin K therapy in critically ill patients, without meaningful impact on clinical outcomes such as bleeding or thrombosis [20].

Taken together, these studies support a selective, evidence-based approach to vitamin K use in cirrhosis, rather than empirical administration based solely on elevated INR.

Similarly, a prospective study by Rivosecchi et al. (2017) [12] evaluated 96 cirrhotic patients (mean MELD score 34) and showed that only a minority responded to intravenous vitamin K. While the median INR decreased by 0.31, approximately 62% of participants achieved less than a 10% INR reduction, and only about 17% exhibited an “effective” response, defined as a ≥30% decrease in INR or reaching INR ≤ 1.5. The study also identified baseline INR as a significant predictor—each 1.0 unit increase in INR doubled the odds of a favorable response—suggesting that effective correction occurs mainly in patients with true vitamin K deficiency superimposed on cirrhosis-related coagulopathy [12].

These findings are supported by Dahlberg et al. (2021), who investigated critically ill patients and reported that vitamin K led to a modest decrease in INR (mean reduction −0.10), with the most noticeable benefit seen in non-cirrhotic patients or those with low body weight [21].

Additionally, Tischendorf et al. (2016) demonstrated that declines in natural anticoagulants such as protein C and antithrombin precede measurable INR elevation in cirrhotic patients, reinforcing the idea that coagulation abnormalities in cirrhosis are multifactorial and not always responsive to vitamin K [22].

On the other hand, several studies have reported modest, early improvements in coagulation parameters following vitamin K administration in select cirrhotic patients, partially aligning with our own findings. Al Sulaiman et al. observed that, in critically ill patients with advanced liver disease, the first intravenous dose of vitamin K led to a statistically significant reduction in INR (median decrease of ~0.63). However, additional doses did not produce further significant improvements, suggesting that the maximum therapeutic benefit occurs early—likely in the setting of actual vitamin K deficiency. Importantly, despite this laboratory improvement, the study found no reduction in bleeding events in patients who received vitamin K compared to those who did not, reinforcing the idea that biochemical correction of INR does not necessarily translate into a clinical benefit [20].

A similar observation was made in cases of acute hepatic dysfunction without established cirrhosis, where Pereira et al. demonstrated that a single 10 mg intravenous dose of vitamin K1 resulted in normalization of prothrombin time within 48–96 h, while oral administration failed to achieve similar results. These findings highlight the importance of parenteral administration in acute settings with presumed or confirmed vitamin K deficiency [23].

Taken together, these studies suggest that vitamin K may be beneficial in specific clinical scenarios, particularly when administered early and in cases where true deficiency is present. However, such improvements should not be overinterpreted as indicators of reduced hemorrhagic risk in the broader cirrhotic population.

The discrepancy between our findings and those of other studies may stem from differences in patient selection and baseline vitamin K status. In our cohort, factors such as cholestasis-induced fat-soluble vitamin malabsorption, inadequate dietary intake, or antibiotic-induced dysbiosis may have contributed to true vitamin K deficiency, thereby explaining the observed INR improvement following supplementation. Clinical literature supports that intravenous vitamin K is more likely to correct coagulopathy in patients with cholestatic liver disease. Moreover, factor V activity can serve as a functional marker: low levels (<50%) indicate advanced hepatic insufficiency and poor response to vitamin K, while normal levels suggest a selective deficiency of vitamin K–dependent factors [22].

In our study, although we did not directly assess levels of factor V or proteins induced by vitamin K absence (PIVKA-II), the observed INR improvement in a substantial proportion of patients indirectly suggests that a vitamin K deficiency may have been present in some cases. This hypothesis is supported by Rivosecchi et al., who found that patients with markedly elevated baseline INR—likely reflecting both impaired hepatic synthesis and vitamin K deficiency—were significantly more likely to experience meaningful INR reductions after intravenous vitamin K administration [12].

This principle is clinically important, as it helps prevent unnecessary treatment in patients unlikely to benefit, particularly given the risk of hypersensitivity reactions associated with intravenous formulations [13]. Furthermore, route and dosage are critical. Studies comparing oral and intravenous administration have shown that only the intravenous route reliably normalizes INR in cases of vitamin K deficiency, particularly in acute or cholestatic liver disease [24]. A typical protocol involves daily administration of 10 mg IV vitamin K for up to three days, with most of the therapeutic effect occurring after the first dose [23].

In our cohort, the majority of INR reduction occurred early, indicating effective correction of the presumed deficiency. Continued dosing yielded minimal additional benefit, suggesting that in responders, repletion was achieved quickly, while in non-responders, coagulopathy was not deficiency-related. These observations, along with inter-study variability in route and dosing (e.g., subcutaneous administration in Saja et al.), may explain discrepancies in reported outcomes and support selective, rather than routine, use of vitamin K in cirrhotic patients.

In cholestatic liver disease, impaired absorption of fat-soluble vitamins, including vitamin K, increases the risk of functional deficiency. Intravenous administration is more effective than oral routes in such cases. The early INR response observed in our study supports this mechanism, possibly linked to malabsorption or poor intake in our cohort [24].

A study by Yoshida et al. [25], which included 548 patients undergoing curative treatment for hepatocellular carcinoma and randomized to receive vitamin K2 (45 mg or 90 mg/day) or placebo, showed that vitamin K2 supplementation did not significantly reduce disease recurrence or improve disease-free survival. This finding contrasts with the hypothesis that vitamin K might have a preventive effect on HCC recurrence, suggesting that the mechanisms of liver carcinogenesis involve factors that are not solely influenced by vitamin K repletion. In our study, we did not follow HCC recurrence, but if vitamin K had a role in preventing carcinoma, it would likely be modest and dependent on the timing of administration, dose, and pre-existing liver and oncological status.

Our findings showed that 43.4% of patients met the therapeutic response criterion at 24 h and 74.6% at discharge, based on a composite definition of response (INR reduction ≥ 30% or INR ≤ 1.5). These proportions are higher than those reported by Rivosecchi et al. [15], who observed an effective response rate of 17.2% using the same definition. This discrepancy may be attributed to differences in patient selection, baseline INR values, timing of reassessment, or underlying hepatic functional reserve. In addition, our cohort showed a higher proportion of patients with compensated cirrhosis (Child-Pugh class A), which may have facilitated a more favorable response to vitamin K administration. These comparative findings suggest that patient-specific factors, especially hepatic synthetic capacity, play a critical role in determining the efficacy of vitamin K therapy in cirrhosis-associated coagulopathy. In our study, changes in coagulation parameters following vitamin K administration were modest and heterogeneous across the cohort. The clearest improvements in INR and prothrombin time were observed among patients classified as Child–Pugh A, suggesting a potentially better response in those with preserved hepatic function. However, in more advanced stages of cirrhosis (Child–Pugh B and C), these improvements were limited, supporting the hypothesis that therapeutic benefit depends on the liver’s residual synthetic capacity. These observations are consistent with prior findings by Meyer et al. [19], who noted that while vitamin K can lead to a reduction in INR, the effect is often minor and may not translate into meaningful clinical benefit in all patients with cirrhosis. Similarly, Al Sulaiman et al. [20] highlighted that a single dose of vitamin K had limited efficacy in correcting coagulopathy, especially in critically ill cirrhotic patients, and emphasized the importance of appropriate patient selection. Our results reinforce the need for an individualized, context-based approach, avoiding empirical use in patients unlikely to benefit.

While modest improvements in liver function tests were observed during hospitalization, these changes raise the possibility that INR reductions may reflect partial hepatic recovery rather than a direct effect of vitamin K alone. This distinction is important, as the synthetic capacity of the liver plays a key role in the production of vitamin K–dependent clotting factors. Previous studies have emphasized that vitamin K administration is unlikely to correct coagulopathy in the absence of a true deficiency, particularly in advanced liver dysfunction. For instance, Smith et al. [26] observed minimal INR correction following vitamin K in patients with decompensated cirrhosis, reinforcing the limited role of supplementation when hepatic function is severely impaired. Similarly, Hambley et al. [27] highlighted that the therapeutic benefit of vitamin K is most evident in cases of cholestasis or malabsorption-related deficiency, not intrinsic hepatic synthetic failure. Our findings align with this, as the greatest INR responses were seen in patients with more preserved liver function.

This study has several limitations. Its retrospective, observational design and lack of a control group limit the ability to directly attribute INR or PT improvements to vitamin K. Other interventions during hospitalization, such as infection management, fluid resuscitation or alcohol cessation, may have influenced coagulation independently. Selection bias is possible, as patients with higher INR or suspected cholestasis may have been more likely to receive vitamin K, while those with severe liver failure were underrepresented. Treatment was not standardized, and dose timing varied between cases. Moreover, vitamin K status was not objectively measured, as plasma levels and PIVKA-II were unavailable, making deficiency only inferable through INR response. The study focused on short-term laboratory outcomes, without assessing long-term clinical benefits such as reduced bleeding or survival. The small, single-center cohort and heterogeneous patient population further limit generalizability and prevented subgroup analysis.

Future studies could better define the profile of cirrhotic patients who benefit from vitamin K administration and assess the clinical significance of this intervention beyond laboratory changes. Additionally, differentiating the therapeutic response based on disease etiology, severity, and signs of cholestasis may help support a more selective and rational use of vitamin K in clinical practice.

## 5. Conclusions

This study highlights the value of a targeted, individualized approach to managing coagulopathy associated with liver cirrhosis. These findings suggest a potential benefit of vitamin K in selected cirrhotic patients with preserved hepatic function or vitamin K deficiency. However, in the absence of a control group and given the retrospective nature of the study, no causal inference can be drawn. The optimal therapeutic approach in correcting coagulopathy associated with liver cirrhosis should be chosen by differentiating between coagulopathy caused by reversible vitamin K deficiency and that caused by irreversible liver dysfunction. Although vitamin K is not universally effective, as correction of coagulopathy may be influenced by multiple factors, its administration should be considered and could improve coagulation parameters in some cases in patients with pre-existing deficiency and should be considered within a broader therapeutic strategy. Its utility lies not in routine use, but in thoughtful, case-based application. Vitamin K is not a general solution for cirrhotic coagulopathy but, when used judiciously, it can make a meaningful difference in carefully selected cases.

## Figures and Tables

**Table 1:** Patient characteristics.

Age (years, mean ± SD)	60.91 ± 14.30
Male/Female (*n*, %)	75 (61.48%)/47 (38.52%)
Urban/Rural (*n*, %)	61 (50%)/61 (50%)
Cirrhosis etiology (*n*, %)	
alcohol	58 (47.54%)
cryptogenic	26 (21.31%)
viral	21 (17.21%)
MASLD	17 (13.93%)
Child–Pugh Classification (at admission) (*n*, %)	
A	63 (51.64%)
B	27 (22.13%)
C	32 (26.23%)
Hospitalization (days, mean ± SD)	10.80 ± 6.00
Death/Discharged (*n*, %)	40 (32.79%)/82 (67.21%)

**Table 2:** INR reductions and effectiveness analysis.

	10% INR Decrease	20% INR Decrease	30% INR Decrease	40% INR Decrease	50% INR Decrease
Reduction in INR (n%) discharge vs. admission	94(77.0)	81(66.4)	56(45.9)	33(27.0)	15(12.3)
Effectiveness analysis * discharge vs. admission	100(90.2)	104(85.2)	91(74.6)	82(67.2)	76(62.3)
Reduction in INR (n%) after the first dose vs. admission	67(54.9)	31(25.4)	13(10.7)	7(5.7)	2(1.6)
Effectiveness analysis * after the first dose vs. admission	85(69.7)	61(50.0)	53(43.4)	51(41.8)	48(39.3)

* Effective was defined as: percentage reduction in INR (>30%) or decrease in INR to absolute value ≤ 1.5, as used in Rivosecchi et al. [15].

## Data Availability

The original contributions presented in this study are included in the article. Further inquiries can be directed to the corresponding authors.

## Abbreviations

The following abbreviations are used in this manuscript:

INR	International Normalized Ratio
PT	Prothrombin Time
MELD	Model for End-Stage Liver Disease
FFP	Fresh Frozen Plasma
MASLD	Metabolic Dysfunction-Associated Steatotic Liver Disease
NASH	Non-Alcoholic Steatohepatitis
PIVKA-II	Proteins Induced by Vitamin K Absence-II
COPD	Chronic Obstructive Pulmonary Disease
